# Inter-limb and inter-agent coordination in an original joint-action game: exploring novel approaches for clinical practice

**DOI:** 10.3389/fpsyg.2025.1514957

**Published:** 2025-03-24

**Authors:** Anaëlle Cheillan, João Milho, Pedro Passos

**Affiliations:** ^1^Faculdade de Motricidade Humana, Universidade de Lisboa, Lisboa, Portugal; ^2^IDMEC, Instituto Superior de Engenharia de Lisboa, Instituto Politécnico de Lisboa, Lisboa, Portugal; ^3^CIPER, Faculdade de Motricidade Humana, Universidade de Lisboa, Lisboa, Portugal

**Keywords:** complex systems, principal component, uncontrolled manifold, degrees of freedom, interpersonal synergy, dimensional reduction, reciprocal compensation, motor exploration

## Abstract

After identifying a need to develop rehabilitation practices inspired from a systems perspective, we designed a joint-action game that involves both inter-limb and inter-agent coordination. The main specificity of our joint-action game lies in the informational and mechanical couplings that exist between the system elements—i.e., between lower limbs at one scale, and between agents at another scale. The present paper aims to investigate whether our joint-action game can foster the emergence of new coordination patterns at both scales, and discuss whether such patterns, if any, could be clinically relevant. Twelve dyads were asked to stand up on an unstable surface (BOSU) and to jointly manipulate a board on which a ball had to roll along a circular path containing target doors. Ball trajectory as well as lower limb and hand kinematics were obtained using an 8-camera motion capture system. Coordination between left and right knee joint angles was assessed through relative-phase and PCA analyses. Inter-agent coordination was evaluated using UCM analyses. The effects of amount of practice and performance on coordination were investigated. At both scales, significant coordination differences were found over practice and across levels of performance. More specifically, left and right knees were constrained to act as a single unit, while interpersonal synergies were observed in trials with better performance. We discussed how the exploration of coordinative solutions, as well as the dimensional reduction and reciprocal compensation among degrees of freedom that our game supports could be beneficially exploited in rehabilitation.

## 1 Introduction

In the late 1980s, the researchers Goldberger and West, respectively specialized in Medicine and Physics, multiplied collaborative studies to develop a dynamical theory of health and disease, which claims that complexity is inherent to healthy biological systems (i.e., functional systems adaptable to perturbations) and that *decomplexification* is a common feature of pathological systems (i.e., non-functional systems vulnerable to perturbations) (Goldberger et al., 1985; Goldberger and West, 1992; Goldberger, 1996). Based on related findings on various pathological conditions, Lipsitz and Goldberger (1992) described this loss of complexity as *“(i) a loss or impairment of functional components, and/or (ii) altered nonlinear coupling between these components.”* While the approach traditionally adopted in clinics use linear models to identify the absence/presence of abnormalities in isolated organ systems, this alternative theory aims to assess the functionality of the body through its level of complexity, where complexity *“arises from the interaction of a myriad of structural units*” (Lipsitz and Goldberger, 1992). This approach and derived theories based on the same conceptual framework (Vaillancourt and Newell, 2002; Stergiou and Decker, 2011) have inspired the contemporary literature to revise the concept of pathology, as well as the techniques used for clinical assessment, prevention and intervention.

### 1.1 Loss of complexity: the example of an ACL injury

A loss of complexity has notably been reported in patients with anterior cruciate ligament (ACL) deficiency, according to the two conditions (i) and (ii) described by Lipsitz and Goldberger (1992) to support this affirmation. First, the loss of functional components in ACL-deficient patients mainly appears as a significant decrease in the number of the mechanoreceptors in the remnants of the ruptured knee ligament, and such a structural loss impairs postural stability at the functional level (Kosy and Mandalia, 2018; Banios et al., 2022). Morphological alterations of these mechanoreceptors have also been reported, with regards to their size (i.e., decreased volume of the stump), their type (i.e., decreased proportion of Ruffini corpuscles) and their denervation (i.e., loss of free nerve endings) (Li et al., 2018; Gao et al., 2010; Denti et al., 1994). In a recent review based on a multi-systems approach (Larson et al., 2021), it was emphasized that many associated quantitative and qualitative degenerations co-occur around the knee structure, and more specifically in terms of muscles (e.g., decreased quadriceps volume, altered fiber type composition, changes in the pennation angle) (Noehren et al., 2016), bone (e.g., decreased bone mineral density of the femur, tibia, patella) (Nyland et al., 2010) and cartilage (e.g., altered articular cartilage thickness and composition, degeneration of chondrocytes, osteochondral lesions) (Frobell, 2011; Johnson et al., 1998).

Second, the loss of complexity in ACL-deficient patients was characterized by alterations in both spatial and temporal patterns. Using fractal dimension analyses as a nonlinear tool to better investigate the influence of an ACL injury on the bone architecture complexity, Birch et al. (2018) reported significantly lower tibial fractal signatures in the ACL-deficient knee compared to the contralateral uninjured knee as well as healthy controls. Altered patterns following an ACL injury were also reported in studies focused on behavioral dynamics (Decker et al., 2011). For instance, in a study assessing quiet stance, the nonlinear dynamics of center of pressure were extracted by recurrence quantification analysis in ACL-deficient patients whose oscillations were found to be significantly more regular than those of the healthy controls (Negahban et al., 2016). In gait studies, entropy analyses revealed excessive periodic patterns in stride-to-stride variability of the ACL-deficient knee compared to the intact contralateral knee (Georgoulis et al., 2006) and to healthy controls (Moraiti et al., 2009), and that this rigidification of gait behavior gets intensified 1 year after rupture (De Oliveira et al., 2019). In summary, fractals and entropy can, respectively, detect spatial and temporal excessive periodicities/regularities in pathological populations while healthy individuals exhibit a functionally organized variability.

Taken together, these findings depict a global loss of complexity in the structural repertoire and behavioral dynamics of the ACL-deficient profiles, which results in a loss of adaptability to perturbations at the functional level.

### 1.2 Assessing and preventing ACL injury from a complex systems perspective

Given the importance of complexity in the functionality of biological systems, epidemiological research should be broadened from a systems perspective with the use of terminologies and analytical tools from the field of nonlinear sciences (Philippe and Mansi, 1998). In this sense, efforts have been made to develop systems-based preventive models for ACL injury. Quatman et al. (2009) reported reductionistic tendencies in ACL prevention research and proposed an alternative conceptual and methodological framework based on the combination of *in vivo*, *in vitro* and silico techniques to better understand the complex relationships between joint biomechanics and joint injury mechanisms. Later, Bittencourt et al. (2016) also encouraged a paradigm shift from a reductionistic to a complex systems approach for sport injuries, illustrating their model with the example of ACL injury: *“Since injury is a complex phenomenon characterized by uncertainties and inherent non-linearity, an ACL injury will emerge when a specific pattern of interaction happens in the presence of an inciting event of a given value. Thus, the best manner to predict an injury is by understanding the interactions among the web of determinants and not the determinants themselves.”* As an additional note to the model they proposed – which encapsulates the notions of *self-organization, emerging phenomenon and enslaving principle* – the authors recommended the use of neural networks, classification and regression trees (CART) and machine learning tools relying on probability computations that can take into account the nonlinear nature of the relationships between injury determinants, rather than the use of linear tools relying on the causality relationship between an isolated risk factor and the occurrence of injury. In a similar vein, a recent study (Yung et al., 2022) provided some illustrative techniques based on the complex systems approach to help clinicians assess readiness for return-to-sport (RTS) after ACL injury. Finally, the incorporation of a systems perspective into rehabilitation allowed the revision of some conventional RTS criteria that reduce the complex alterations following ACL injury to a one-leg problem (e.g., limb symmetry indexes) (Larson et al., 2021; Wellsandt et al., 2017; Benjaminse et al., 2018).

### 1.3 Designing ACL rehabilitation from a complex systems perspective

Whereas the benefits of using a systems perspective are getting more recognized in the depiction of structural and functional consequences following ACL injury as well as in the related prevention research, only a few studies have considered integrating them into interventions in ACL rehabilitation. Restoring complexity in ACL-deficient patients could be done through exercises that (1) recruit different body structures and reinforce the coordination between them and/or (2) induce variability in terms of practice contexts and movement solutions, so that the behavioral repertoire is enriched and hence enhances adaptability to perturbations.

Dischiavi et al. (2020) used biomechanical knowledge on force dissipation over multiple joints through kinetic chains to suggest an improved version of an ACL rehabilitation exercise, where trunk, pelvis and hip muscles are involved in a three-planar manner to control the dynamic knee valgus (DKV), often involved in ACL injury. This effort into engaging the body “as a whole” in ACL interventions is also present in Benjaminse et al. (2018) study, where the unilateral cueing *“do not let your ACL knee roll inward when landing”* is replaced by the task *“reach both knees towards the cones in front of you when landing.”* The authors emphasized in a review (Gokeler et al., 2019) how improving postural stability in ACL patients was better achieved when providing instructions related to the task (e.g., *“try to minimize movement of the bars on the balance board you are standing on”*) compared to instructions related to specific limb segment positions. They also mentioned that other rehabilitation methods based on motor learning knowledge should be further investigated in ACL research, including the Differential Learning (DL) method.

Based on the theory of dynamical systems, the DL method considers learning as a self-organizing process where amplifying stochastic perturbations destabilizes the body system and encourages it to explore a large diversity of solutions for reaching the task goal (Schollhorn and The, 2012). In a recent study (Ghanati et al., 2022), the DL method – inferring variability in both task goals and environmental conditions – was shown to be more efficient in reducing ACL risk injury compared to methods based on presenting external targets or on prescribing the ideal pattern of movement with demonstration, imitation, repetition and correction. Finally, Mohammadi et al. (2021) also highlighted the importance of variability for learning. More specifically, they recommended to guide learners to adapt to manipulated task and environmental constraints (i.e., Constraints-Led Approach, CLA) rather than adding random and imposed (not self-regulated) variability during exploration (i.e., DL).

### 1.4 The joint-action hypothesis

The previous paragraph reviews how the problem of degrees of freedom (DoF) posed by Bernstein (1967) has recently been applied in rehabilitation. Instead of prescribing patients a gold standard movement to reproduce, clinicians are encouraged to promote variability in their rehabilitation programs so that patients can better explore the task solution space when re-learning a motor skill. This active exploration leads to an enhanced ability to cope with a greater number of DoF, and the enrichment of the movement coordination repertoire is functional as it allows (i) the emergence of adaptative solutions regarding post-injury individual constraints, and (ii) an increased adaptability to balance perturbations with a reduced risk of re-injury (Mohammadi et al., 2021).

Because systems-based therapies are still in their infancy in rehabilitation, we feel the need to create an experimental task where coordination between system elements could be assessed. With this purpose, we developed a joint-action game that consists in standing on an unstable surface (i.e., the widely-used BOSU balance trainer) while jointly manipulating a board on which a marble rolls along a circular target. This original game is an exploratory task of which the design was inspired by the need of testing a novel approach for clinical practice (i.e., functional challenge) and based on four speculations (i.e., technical solutions). First, the recreational properties inherent to games should increase motivation in the re-learning process. Second, it can be viewed as a functional task, since joint actions are commonly observed in everyday life, whenever two or more agents coordinate their actions to reach a common goal (e.g., shaking hands, navigating on a crowded street, but also, for more similarities, lifting and moving the dinner table with your guests). Third, our joint-action game encourages the players to explore various movement solutions as they try not to fall while coordinating their actions towards a supra-postural task goal; in other words, it should expand their behavioral repertoire and adaptability. Fourth, one of the main guidelines of the manufactory process of our joint-action game arises from Bernstein’s hypothesis (1967) (Bernstein, 1967) that let us think that informational and mechanical couplings in this task should reduce the great dimensionality of DoFs existing at both inter-limb and inter-agent scales – i.e., the system elements should be constrained to behave as a single unit through the couplings implied by our game. Such a dimensional reduction may have a particular clinical interest as it should lead the uninjured and injured elements to re-learn how to move as a whole. Furthermore, these low-dimensional units may be characterized by a reciprocal compensation between the systems elements, which also might be clinically beneficial.

To clarify, our game aims to promote both the exploration and structuring of redundant DoF (i.e., where multiple movement solutions can achieve the same task goal). While movement variability (emerging from redundancy) should allow patients to discover adaptive solutions in response to balance perturbations, dimensional reduction should foster coordination efficiency by organizing these redundant DoF into stable, low-dimensional, functional units (or synergies). The major novelty in this study is most likely how our joint-action game paves the way for investigating whether the players’ actions can be compensated when performing the task (i.e., whether interpersonal synergies can emerge from practice) and thereby lead to postural reorganization. When both dimensional reduction and reciprocal compensation occurs among the system elements, the term *“synergy”* can be used (Riley et al., 2011) and will be mentioned in this sense in the present paper. After having designed the functional and technical specifications to build the game, the natural next step was to scientifically evaluate its potential. For that purpose, the present paper aims to answer two research questions:

1. How does inter-limb coordination emerge from our joint-action game?
2. How does inter-agent coordination emerge from our joint-action game?

We hypothesized that the effects of practice of our joint-action game would manifest as (1) the emergence of novel lower-limb coordination patterns, where both left and right limbs should be jointly coupled as a single unit, and (2) the emergence of interpersonal synergies (as described by both dimensional reduction and reciprocal compensation properties), where participants’ movements should be reciprocally compensated to successfully perform the game. Finally, the clinical relevance of the coordination patterns emerging from our game, if any, will be discussed.

## 2 Materials and methods

### 2.1 Participants

The study involved 24 university students (10 males, 14 females; mean age: 21.83 ± 2.12 y-o.). Participants were randomly assigned into 12 dyads. Sample size estimation was based on *F*-tests (ANOVA, Repeated Measures, Within Factors) for a group with 2 measurements, using parameters such as an effect size of 0.40, an alpha error probability of 0.05, a beta error probability of 0.20 (yielding 80% power), and a correlation between measures of 0.50. These parameters were determined using the G*Power software (Universität Düsseldorf, Germany). All participants confirmed no prior history of lower limb injury, surgery, or any condition that could impair postural stability. Informed consent was obtained before the experiment began, and the study was approved by the Ethics Committee of the faculty where the research was conducted.

### 2.2 Experimental design

The experimental session consisted of 30 trials. In each dyad, both participants stood face-to-face barefoot on two unstable surfaces (BOSU) while using a supination grip to jointly manipulate a board over which a ball rolled. The board contained a 10-cm wide target path defined by two circles (inner circle radius: 20 cm; outer circle radius: 30 cm). Four equidistant doors were added along the target and defined by paper pins on both circles, at the nearest and furthest target points from both participants. Similarly to road markings, the midline of the target path was also drawn as a landmark.

**Figure 1:**
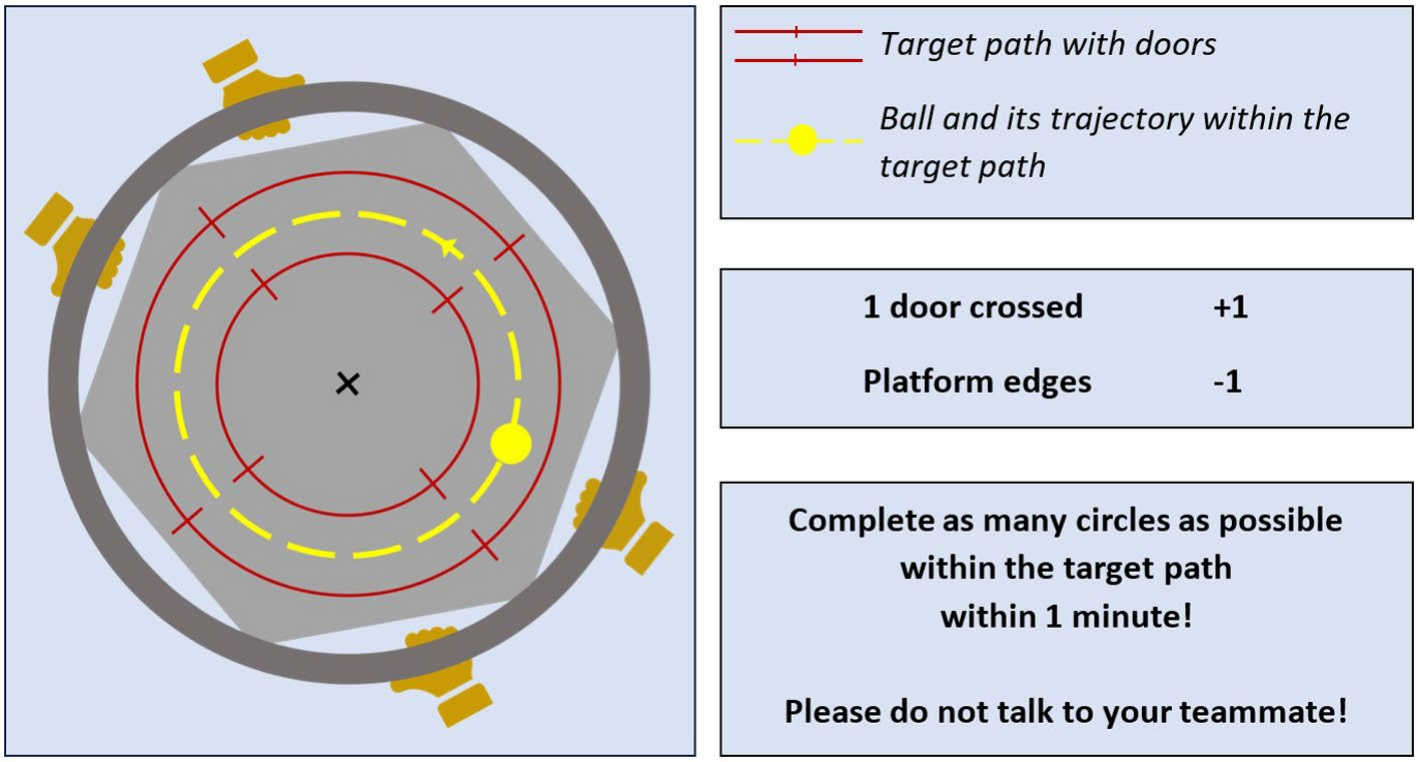
Instructions given to the participants before the ball-and-board game starts. For each dyad, one participant counted the number of times the ball rolled through a door (i.e., number of crossings), while the other participant counted the number of times the ball bumped into the board edges (i.e., number of penalties). Board movements were tracked using markers on the board’s center and on the participants’ hands (distal wrist crease) fixed on the board handle.

The task goal provided to the participants was to jointly control the ball trajectory on the board, in such a way that the ball completes as many circles as possible within the target path within 60 s (Figure 1). In each trial, one participant was instructed to count the number of crossed doors (i.e., 1 point was gained at each door crossed by the ball), while the other one counted the number of penalties (i.e., 1 point was deducted at each collision of the ball with the board’s edge). All along the experiment, the current best score (i.e., number of crossings – number of collisions) was reminded to the dyads to keep them engaged into the game. Please note that performance scores were later computed by the experimenter using objective measures (as described further below), while the scores counted by participants were only used for motivational purposes. If the ball fell out of the board or if one of the participants fell out of the BOSU, the trial was performed again. No verbal communication about game strategy was allowed during the experiment.

Following the recommendations given by participants of the pilot test, we decided to split the session into two 15-trial sets (i.e., Set 1 and Set 2) which were separated by a 5-min break to avoid fatigue. To ensure that the game instructions were clearly understood, the participants freely played for less than 3 min before the session was recorded.

### 2.3 Apparatus

Our experimental device can be defined as a ball-and-board system (mass: 2.5; dimensions: 114 × 114 × 8 cm), which is composed of a hexagonal board (long diagonal: 96 cm, made from polyurethane). The board is connected to a ring-shaped handle constructed from a 25-mm polyethylene pipe, designed for easy gripping by participants. Six rigid PVC pipes serve as connectors between the board and the handle, and six polystyrene rods are affixed along the board’s edges to act as a ball-stop system. The primary criteria for material selection were to ensure the ball-and-board system remained lightweight yet rigid, given its relatively large size. The ball rolling over the board is a reflective marker (diameter: 12.7 mm). Another reflective marker was positioned at the board’s center. The unstable surface on which each participant stands is provided by a BOSU ball, which features an inflated rubber hemisphere attached to a rigid platform. To challenge even more postural stability, the rounded side of the BOSU was placed on the ground, with the participants standing on its flat, rigid surface.

Data were collected using the OptiTrack motion capture system (NaturalPoint, Corvallis, Oregon) equipped with 8 cameras, at a sampling frequency of 60 Hz. Ball motion relative to the target drawn on the board was obtained using the ball reflective marker as well as markers placed on the board’s center and extremities. More precisely, the reflective markers defining the board extremities were defined by the participants’ hands fixed on the board handle. Hand markers were placed on the distal wrist crease, as it is an anatomical landmark easily identifiable by palpation, and tight specific garment were used to minimize inaccuracy in kinematic measurements. This methodological choice was validated during pilot tests, as wrists single-markers were never hidden by the ball-and-board system, and they were sufficient to obtain the single-plane data (i.e., vertical plane of motion) required to address our research question at the inter-agent scale.

Regarding measurements at the inter-limb scale, four rigid bodies with three mounted markers were attached on each thigh and each shank to obtain knee joint angle. Rigid bodies may offer several advantages compared to anatomical markers. As only clusters are tracked, the former’s quality relies less on the marker placement. Implementing redundant markers enables us to overcome the missing marker problem that may have occurred with single anatomical markers (e.g., hidden by the large, opaque board held by participants). The techniques using clusters also identify body segment location in relation to a plane and, consequently, reduce the effects of soft tissue artifacts on segment pose. These rigid bodies – worn by only one participant in each dyad – allowed analyses at the inter-limb level. At this level, the focus of a single participant can be viewed as a clinical setting simulation, where only the ACL-deficient patient in a healthy-pathological dyad would have worn the lower limb markers.

### 2.4 Data processing and analysis

Three-dimensional movement data were recorded and labelled using the software Motive: Body 2.1.1. Data were exported at 28 Hz to Excel and then processed in MATLAB (version R2023b, MathWorks Inc., USA). This sampling frequency was selected based on recommendations from studies investigating inter-limb or inter-agent coordination during a supra-postural task or a joint-action task performed on an unstable surface (Bardy et al., 1999; Montull et al., 2020). The ball trajectory relative to the target doors in the board coordinates system was obtained using the markers on the ball and board. In each trial, the first 3.5 s were trimmed to account for the ball not always starting its motion immediately, ensuring a continuous 60-s trajectory (i.e., 1,680 datapoints). The number of doors crossed by the ball was analyzed as a performance variable.

Before running the analyses described below, a linear filling algorithm was used to solve occasional missing data issues (i.e., Matlab *fillmissing* function with the *linear* argument). Because the sample frequency was relatively low (i.e., 28 Hz) and because no derivative of variables was analyzed, no filter was applied to the datasets to avoid removal of potentially precious information from the signals recorded.

#### 2.4.1 Inter-limb coordination

Knee joint angles were used to study coordination between the lower limbs. This methodological choice was motivated by our interest in examining whether maintaining balance on the BOSU during the joint-action game would challenge knee–knee coordination, as this could offer insights for replicating the study in a population with knee injury. Knee joint angles were obtained using the rigid bodies on thighs and shanks (as described above) and calculating the quaternion product for thigh and shank segments (i.e., using Matlab functions *quatmultiply* and *quatconj*). At each frame of time series, angular position differences between left and right knee angles were calculated. For that purpose, Hilbert transform was applied to compute phase angles, which were normalized to a 0–180° range. Phase angles were categorized into nine 20° relative-phase regions. For each dyad and each trial, the percentage occurrence of phase angles in each region was calculated and used to analyze the coordination patterns between the left and right knees. To examine whether these coordination patterns were adopted in a significant proportion of the trial, 1,000 relative-phase time series of corresponding sample length (60 s) and frequency (28 Hz) were randomly created, thereby generating 1,000 random relative-phase distributions. The 950^th^ largest value – 12.3% – was employed as a statistical threshold value. This methodology is consistent with conventional statistical practices, where the goal is to determine whether an observed outcome is significantly different from what would be expected under the null hypothesis (i.e., no significant coordination pattern). This establishes a significance level of 5%, as it identifies the upper 5% of the distribution. Hence, any observed relative-phase region of which the occurrence was greater than 12.3% reflected a coordination pattern that was deemed to be significantly prevalent within the trial, as it exceeds the threshold determined by the 95% of the random samples. In applying this methodology, we followed the procedures outlined by Nalepka and colleagues in their two-player experiment (2017) (Nalepka et al., 2017).

The average relative-phase distribution across dyads and trials was inspected. For all dyads, Pearson’s correlation analyses between average performance scores and relative-phase distributions across trials were also used to give insight into the functionality of the coordination patterns adopted. Cohen’s guidelines (1988) (Cohen, 1988) were followed to interpret correlation strength, where the coefficient threshold value *r* = 0.5 implies high correlation.

Subsequently, a principal component analysis (PCA) was conducted using a MATLAB function to identify coordinative solutions among all dyads. PCA was applied to the mean-centered relative-phase data of the knee joints. By mapping original data into a space where the axes, or principal components, represent the directions along which the data distribution spreads most, PCA provides a global picture of coordination resulting in a reduction of dimensionality, rather than a set of relative phase measures between joints (Daffertshofer et al., 2004; Forner-Cordero et al., 2005). A two-component PCA was performed after finding that retaining two relative-phase regions could explain at least 95% of the variance in knees data. For each dyad, PC score is defined by the linear combination of components PC1 and PC2 explaining the maximal fraction of data variance. Graphically, it refers to the coordinates of a datapoint in the PCA space. PCA was conducted for each dataset (Set 1 and Set 2), and PC scores for individual dyads were computed by projecting dyad-specific data onto the principal components. This approach ensures consistent component axes across dyads within the same set.

Statistical analyses to compare the PC scores obtained in Set 1 and Set 2 were conducted using the non-parametric Wilcoxon’s test, since the normality assumption was violated according to Kolmogorov–Smirnov’s test. Wilcoxon’s tests compared the differences between sets in PC1 scores and in PC2 scores separately. Because PC1 and PC2 represent, respectively, the *x-* and *y-*coordinates of a global coordinative solution (i.e., datapoint in the two-dimensional PCA space), a Wilcoxon’s test was also used to compare the Euclidean distance between each datapoint of coordinates (PC1, PC2) with null distance (i.e., trajectory in the PCA space). This analysis allowed us to better assess the evolution of lower-limb coordination patterns from Set 1 to Set 2. The choice of this non-parametric test was supported by Mardia’s test, which indicated that the covariance matrix (PC1, PC2) did not describe a multivariate normal distribution. The statistical *p*-value threshold was set at *p* < 0.05.

To assess the relationship between inter-limb coordination and performance, Pearson’s correlations were computed between the percentage distribution of relative phase across nine regions and corresponding performance scores. This yielded nine coefficients, each indicating the strength of association between performance and relative phase distribution, offering insights into how coordination patterns relate to task performance. The statistical *p*-value threshold was set at *p* < 0.05.

Due to issues with rigid bodies, Dyad 4 and five trials from Dyad 2 were excluded from inter-limb analyses.

#### 2.4.2 Inter-agent coordination

This second part of analyses aimed to investigate whether reciprocal compensation occurred with practice between the two agents’ movements, which is consistent with interpersonal synergies formation on our joint-action game. For this purpose, the uncontrolled manifold (UCM) method was employed. To address Bernstein’s problem of DoF (1967) (Bernstein, 1967), the UCM was introduced as an analysis to investigate how the redundant human motor system with its large number of DoF was organized to be more controllable in a motor task, i.e., how these DoF could co-vary so as to stabilize a *performance variable* (PV) in a motor task. The method was later extended to the concept of synergy – i.e., functional grouping of structural elements (or DoF) which are temporarily constrained to act as a single coordinated unit (Kelso, 2009) – and at the interpersonal scale in dyadic tasks (Black et al., 2007; Romero et al., 2015). These task-relevant elements that can be functionally coupled into synergies to stabilize a PV are here called *elemental variables* (EVs).

The UCM is a subspace built on the variance of EVs and can be described as a geometrical subspace that contains all possible combinations of EVs values that stabilizes the PV around a reference value, i.e., for which the task is successfully achieved. The variance of EVs is then divided into two components: the variance *V_UCM_* along the UCM—which stabilizes the PV at its reference value—and the variance *V_ORT_* orthogonal to the UCM—which leads to a deviation of the PV from its reference value (and thus from task success). When the ratio *V_UCM_/V_ORT_* (also called *UCM score*) is greater than 1, the EVs variability is functional as it essentially leads to a stabilization of the PV, which is consistent with synergies formation.

To test the emergence of interpersonal synergies (and therefore reciprocal compensation between agents) in our joint-action task, the first step was to select UCM variables that would verify two conditions for the applicability of the method: (i) the relationship existing between the PV and the EVs is linear, and (ii) the PV is aimed to be stabilized. The PV selected was the height of the board using the marker on the board center, as moving the board to extreme heights can prevent the players from perceiving the ball and can lead to their destabilization. In other words, stabilizing the board center at a certain height is relevant for task performance. A linear regression analysis between the board center height and the amount of practice (i.e., trial number) supported us to select this PV, as presented in the Results section. The PV average value obtained along each trial was used as a reference value. The EVs selected were the height of the two points on the ring handle that are in front of the participants—i.e., for each participant, the height of the middle point between their hands. They were called “hands height” for simplification. The board center height can be directly given by the participants’ hands heights because the three points are positioned on the same line on a non-deformable rigid board. One method limitation may be raised as the wrist marker Z-positions do not exactly equate to the positions of the board extremities; however, the board center Z-position was also slightly elevated at a corresponding height of the wrist markers and the wrists’ rotations were restricted as the hands were fixed on the ring handle. In other words, the board center point and the participants’ hands points were considered to be on the same plane. Please note that this methodological choice was retained to minimize the number of markers in an experimental setup that can easily compromise the quality of data collection. In sum, the UCM analysis allowed us to inspect whether the participants’ hands movements in the vertical plane were reciprocally compensated to stabilize the height of the board they jointly held.

Once the PV and EVs selected, a within-trial UCM analysis was performed using the methodology presented by Passos et al. (2018, 2020), itself based on the linear multiple regression method employed by Klous et al. (2010). The detailed procedure used for UCM computations is described in Appendix A. For each trial of each dyad, the UCM score—which indicates synergy formation when greater than 1—was calculated.

For each dyad, UCM scores were averaged across the first set and the second set of the experiment and compared using a paired *T*-test. Normality and homoscedasticity assumptions were tested beforehand, using the Kolmogorov–Smirnov’s and Levene’s tests, respectively. Paired *T*-test were also performed to compare the UCM scores obtained in lower and higher performance. Groups of performance—i.e., *Low Performance* versus *High Performance*, were defined according to the median score obtained from the matrix regrouping the scores of all dyads and trials. In both set and performance group comparisons, significant differences in the UCM scores were reported when *p* < 0.05. In addition, Pearson’s correlation analysis was performed to further explore the relationship between the performance scores and UCM scores at the first and second set, as well as over the whole experimental session. As mentioned above, Pearson’s coefficient threshold values *r* = 0.3 and *r* = 0.5, respectively, indicated medium and high correlations (Cohen, 1988). Again, the significance threshold *p* < 0.05 was used.

Due to missing wrist data, Dyad 10 was excluded from the UCM analyses relating to inter-agent coordination.

## 3 Results

The present section addresses how coordinative solutions are influenced by the amount of practice and related to task performance at (i) inter-limb and (ii) inter-agent scales.

### 3.1 Inter-limb coordination

**Figure 2:**
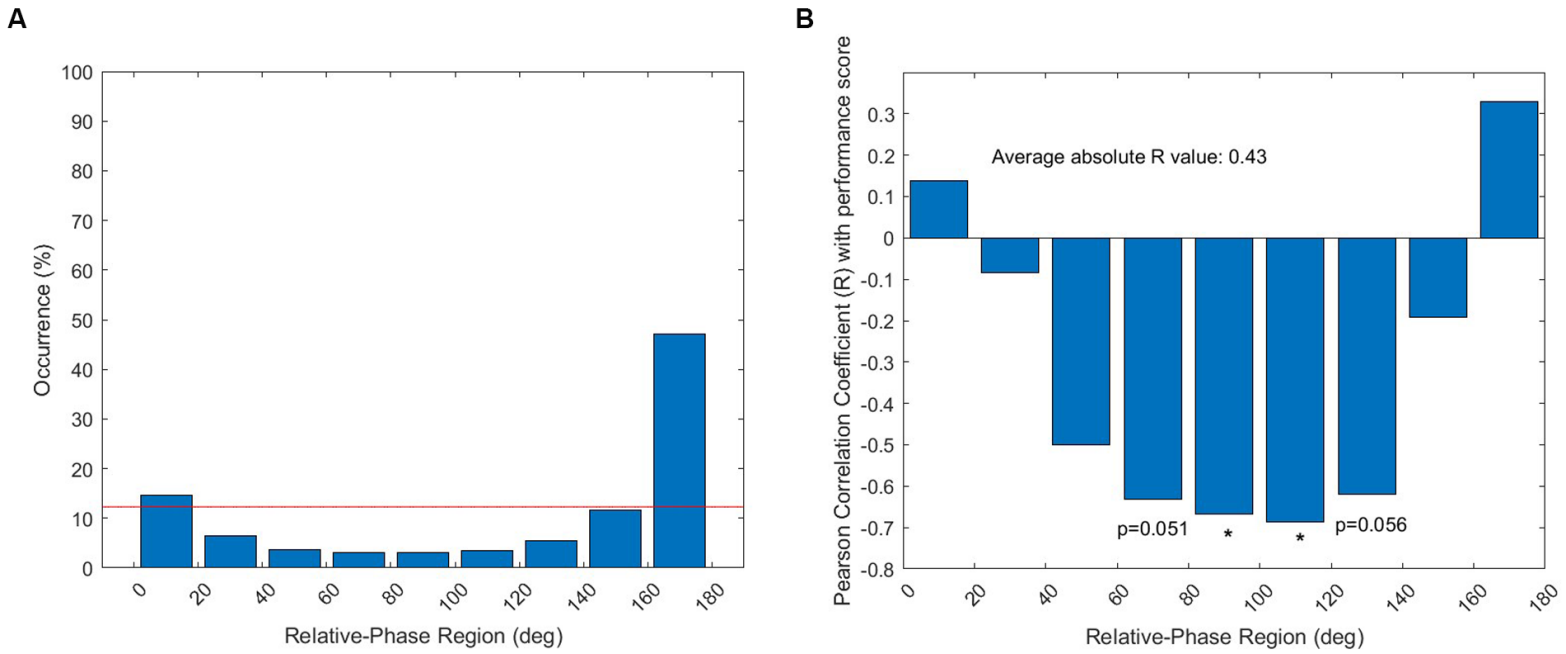
Representation of inter-limb coordination based on relative-phase distribution. **(A)** Occurrence (%) of each of the nine 20° relative-phase regions in all dyads and trials. The red line represents the occurrence threshold (i.e., 12.3%), above which the relative-phase region is deemed to be significantly prevalent. **(B)** Pearson’s correlation between performance and relative-phase distribution in all dyads and trials. For each relative-phase region, Pearson’s coefficient *r* is calculated. The average absolute value of *r* across all relative-phase regions indicates the overall correlation strength between performance and coordination. (*p* < 0.05*).

Coordination between left and right knee angles is illustrated in Figure 2 through nine 20° relative-phase regions. Figure 2A depicts the average relative-phase distribution across dyads and trials. Overall, relative phases from 0 to 20° and from 160 to 180° were significantly prevalent during the experimental session (i.e., percentage occurrence greater than 12.3%). In other words, *in-phase* and *out-of-phase* coordinative modes were mainly adopted, while in-between coordination patterns were not found to be significant.

The correlation between coordination patterns and performance is presented in Figure 2B. Overall, it was found that 18.5% of the variability in the relative-phase distribution was associated with the performance variability (i.e., │*r*│ = 0.43). In-phase and out-of-phase behaviors were positively correlated with performance (i.e., *r* > 0 found in both 0–20° and 160–180° regions), while in-between coordination patterns were negatively correlated with performance (i.e., *r* < 0 found in the other intermediate regions). More specifically, the strongest negative correlations were found to be significant in the regions from 80° to 120° (*p* < 0.05) and tended to be significant in the bordering regions. In these regions, the *r* coefficient values comprised in the range – 0.7 < *r* < − 0.6 indicated a high correlation (Cohen, 1988). An increase in the occurrence of these intermediate patterns were thus significantly linked to a large decrease in performance.

**Figure 3:**
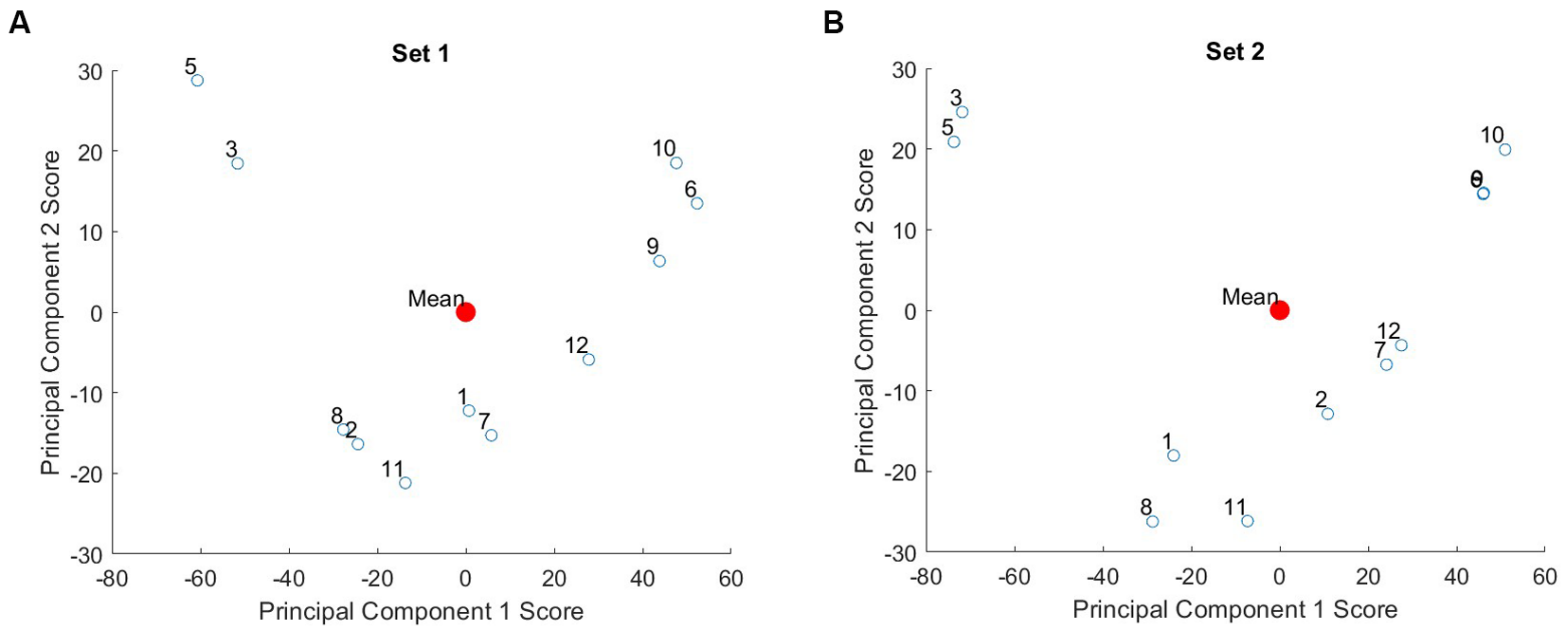
PCA results for all dyads (but Dyad 4; numbered circles) in Set 1 **(A)** and Set 2 **(B)**. Out-of-phase pattern primarily affects the position of data along the PC1 axis, while in-phase pattern primarily influences the position of data along the PC2 axis.

The results from the two-component PCA analysis on the relative-phase distribution of each dyad are illustrated in Figure 3 over the first set (Figure 3A) and the second set (Figure 3B). PC1 – which captured 82.11% of the variance of the data – was mostly influenced by the 160–180° region (i.e., out-of-phase) and PC2 – which captured 16.13% of the variance of the data – was mostly influenced by the 0–20° region (i.e., in-phase). For all dyads (but Dyad 4), the PC scores are graphically represented by the coordinates of the data points in the space defined by PC1 on the *x-*axis and PC2 on the *y*-axis. In complement, the performance scores obtained by all dyads (but Dyad 10) in Set 1 and Set 2 are given in Appendix B (total median score: 30) to give more insight into the clusters of different coordinative solutions.

Wilcoxon’s tests revealed that neither PC1 scores nor PC2 scores were significantly different between the first and second sets (*p* > 0.05). However, the PC scores (i.e., as defined by the combination of PC1 and PC2) were found to be significantly different between the two sets when comparing the actual dyads’ trajectory with a null trajectory in the PCA space (*W* = 66, *p* < 0.001). In other words, neither out-of-phase nor in-phase patterns were significantly more/less present in the coordinative solutions adopted with practice; however, the effects of practice were significant when considering the overall coordination as captured by the combination of PC1—mainly influenced by out-of-phase pattern—and PC2—mainly influenced by in-phase pattern.

Pearson’s analyses between performance and PC scores at each set only revealed significant correlations in PC2 score in Set 1 (*p* < 0.05, *r* = 0.68) and in Set 2 (*p* < 0.01, *r* = 0.87). In other words, a better performance was significantly associated with a very high (Cohen, 1988) increase of the in-phase manifestation in both sets.

Finally, clusters of different coordinative solutions may be identified across dyads (i.e., groupings of datapoints in the PCA space) and may be cautiously regarded in terms of performance – regarding the results described above on the correlations between performance and relative-phase distribution as well as between performance and PCA score. Apart from Dyad 3 and Dyad 5 who distinctively adopted a specific coordinative solution described by a weak out-of-phase component with a strong in-phase component, it seems that dyads with lower performance and dyads with higher performance may be identified within two separate clusters (Figure 3 and Appendix B). More specifically, dyads with lower performance seem to select solutions which are characterized by a relatively weak in-phase component as well as an out-of-phase component that is not considerable either. In contrast, dyads with higher performance seem to demonstrate coordinative solutions that combine both in-phase and out-of-phase components. Furthermore, it seems that some dyads such as Dyad 2 and Dyad 7 – whose performance increases – tend to get away from the cluster where neither in-phase nor out-of-phase are important and to get closer to the cluster characterized by a combination of strong in-phase and strong out-of-phase components at the second set of the experiment.

### 3.2 Inter-agent coordination

**Figure 4:**
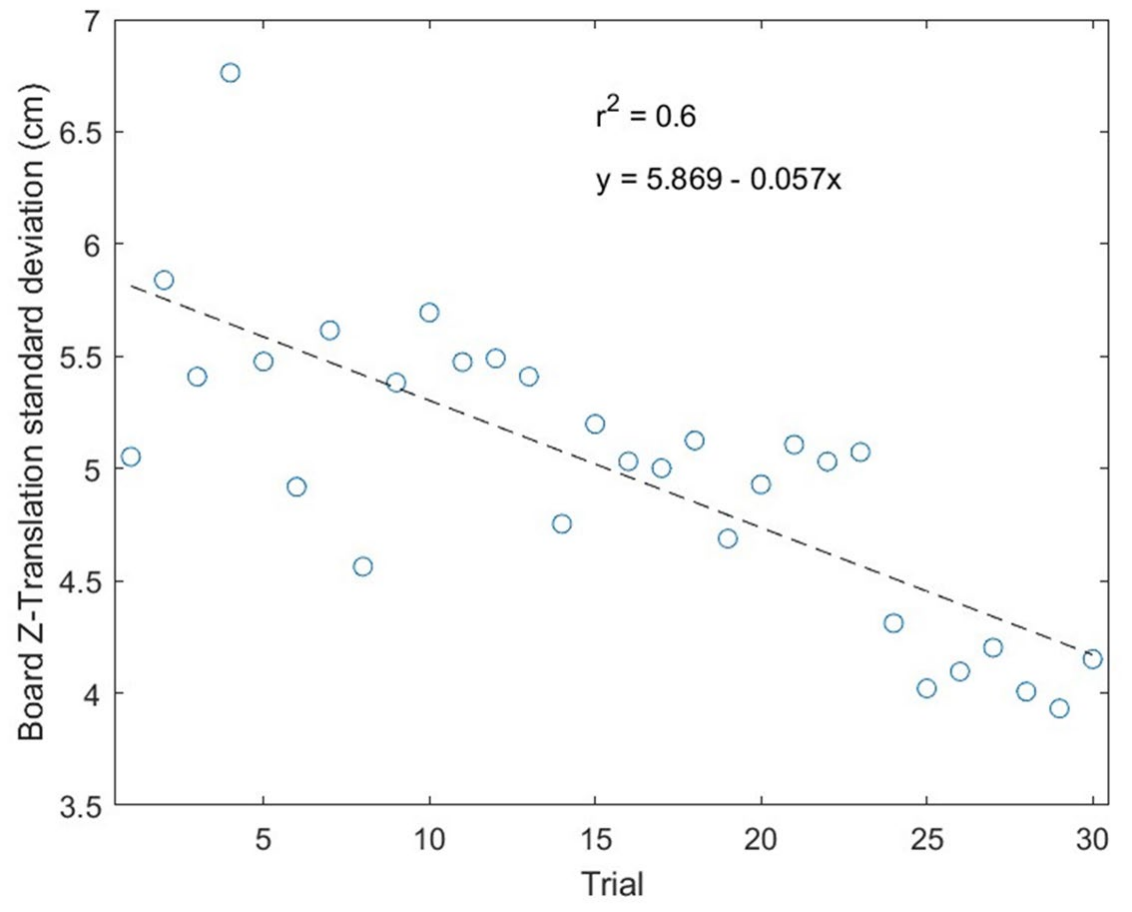
Evolution of the within-trial standard deviation of board center height along the 30 trials of the session. Average values were computed over all dyads (but Dyad 10). Association between the board height standard deviation and the trial number is predicted by the regression equation fitted on the right panel of the figure: *y* = 5.87–0.06 *x*. *R^2^* = 0.600.

Linear regression analysis was performed to test whether the amount of practice (i.e., trial number) significantly predicted the standard deviation of board center height (Figure 4). The regression model (*y* = 5.87–0.07 *x*) was statistically significant (*F* = 41.98, *p* < 0.001). 60% of the variation in the board center height variability was associated with the variation in the amount of practice (i.e., *R^2^* = 0.600), which can be interpreted as a strong correlation according to Cohen’s guidelines (1988) (Cohen, 1988). Because the stabilization of the board center height was a significant effect of practice, this UCM candidate performance variable was therefore selected.

**Figure 5:**
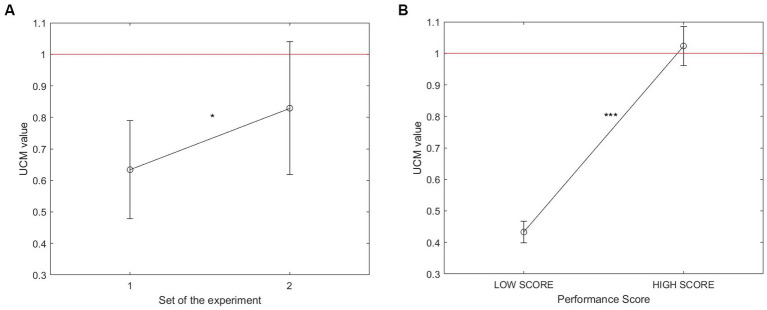
Comparison of the average UCM ratio (across dyads) between Set 1 and Set 2 **(A)** and between *Low-Score* and *High-Score* trials **(B)**. (*p* < 0.05*, *p* < 0.001***). The red line represents the ratio threshold UCM = 1, indicating synergy formation when exceeded.

The following results are obtained from a UCM analysis used to investigate how the dyads’ players coordinate their hand movements in the vertical plane (EVs) to stabilize the board center height (PV). The evolution of the UCM ratio (indicative of the presence/absence of synergy and inherent reciprocal compensation between the players) with practice and performance is revealed by paired T-tests and presented in Figure 5.

Figure 5A shows that the UCM ratio value (i.e., UCM score) significantly increased from Set 1 to Set 2 (*t* = −2.62, *p* < 0.05). Despite this increase, no interpersonal synergy was found on average in the second part of the session (i.e., UCM < 1). Nevertheless, some variability in the data suggests that synergy might have been present in a few trials of the second set (i.e., upper extremity of the error bar above the threshold UCM = 1).

In Figure 5B, the trials were divided into trials with lower performance (i.e., score inferior to the median score) and trials with higher performance (i.e., score superior to the median score). Out of a total of 330 trials (i.e., 11 dyads, each of them performing 30 trials), a total of 15 trials where the score equals to the median score was excluded. The UCM ratio value (i.e., UCM score) was found to be significantly greater in high-score trials compared to low-score trials (*t* = −8.28, *p* < 0.001). On average, inter-agent synergies emerged in trials with higher performance (UCM > 1).

**Figure 6:**
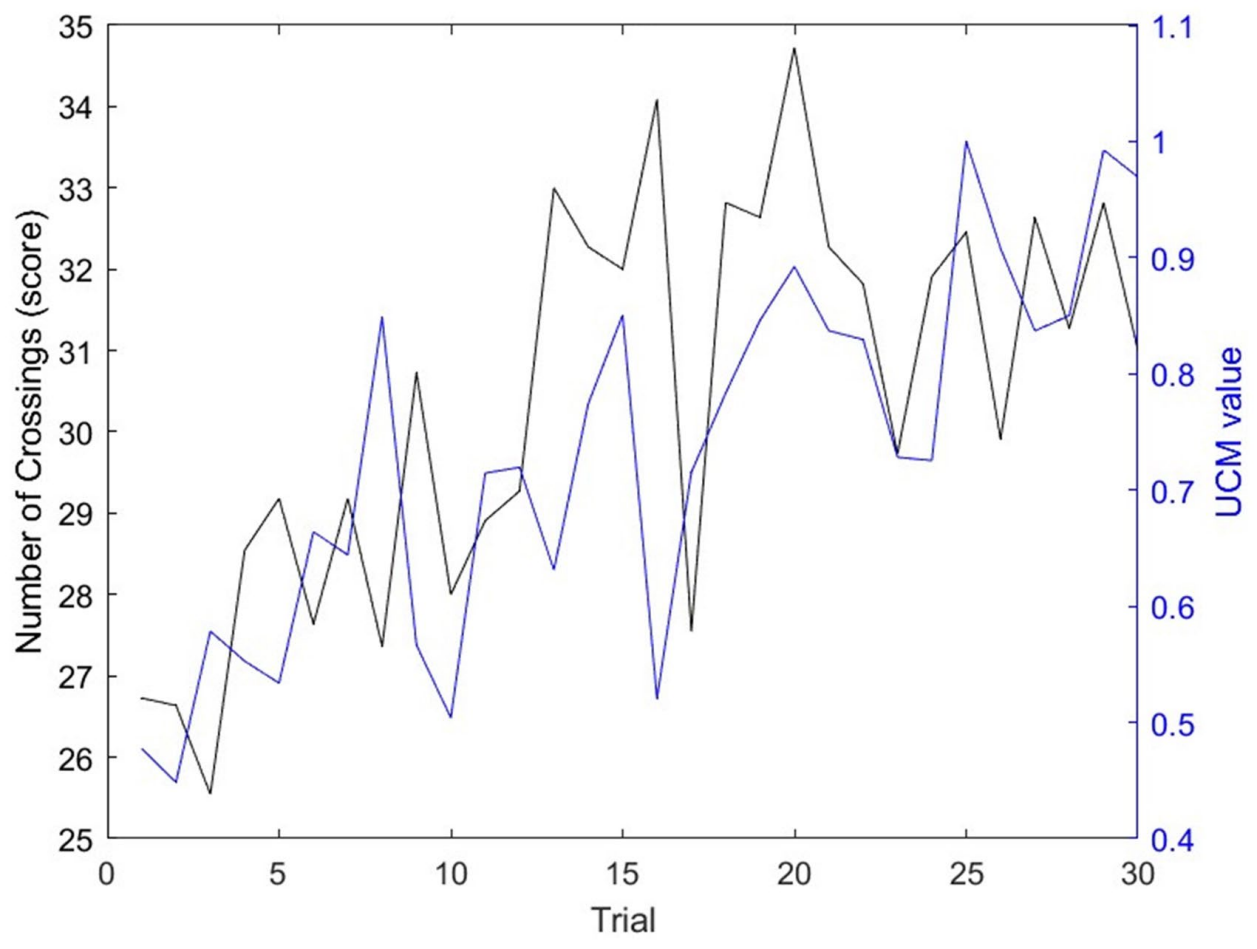
Average evolution (across dyads) of performance (i.e., number of crossings; black) and interpersonal coordination (i.e., UCM value; blue) over the 30 trials of practice. Pearson’s analyses revealed a significant positive correlation between performance scores and UCM scores and this correlation tended to be high (*p* < 0.001, *r* = 0.40).

The relationships between practice, performance and interpersonal coordination are finally depicted in Figure 6. In both sets, performance and UCM values were significantly and positively correlated (i.e., Set 1: *p* < 0.0001, *r* = 0.46; Set 2: *p* < 0.001, *r* = 0.34). Over the whole session, the increase in performance was also significantly correlated with the increase in UCM value (*p* < 0.001, *r* = 0.40). Overall, 16% of the performance increase was significantly related to the UCM increase (i.e., *r*^2^ = 0.16).

## 4 Discussion

Our game was designed from hypotheses formulated on the basis of a systems perspective of Motor Learning—using the principles of Ecological Psychology and Complex Dynamical Systems theory—thereby both informational and mechanical couplings in our game should constraint both lower limbs at one scale and both agents at another scale to act as a single low-dimensional unit. Interpersonal synergies—as characterized by both dimensional reduction and reciprocal compensation properties—were also expected to emerge through our joint-action game. From a clinical standpoint, the design of our game, grounded in these hypotheses, was driven by the motto “re-learning perception-action as a whole.” The purpose of the present study was to investigate whether our game could lead to the emergence of new coordination patterns at both inter-limb and inter-agent scales and to discuss whether such new patterns—if any—could be clinically relevant.

### 4.1 Lower-limb control: dimensional reduction and exploration of the dynamical landscape

Inter-limb analyses allowed the identification of two main coordination patterns between left and right knee joint angles: out-of-phase (as defined by the region 160–180°) and in-phase (as defined by the region 0–20°). Indeed, these phase relations were found to be significantly prevalent among the nine 20° regions analyzed and they could explain more than 95% of the variance in the knee relative-phase data. Despite the absence of significance, out-of-phase and in-phase were also the only coordination patterns positively correlated with performance. The complementary PCA analysis also revealed that an increase in the secondary principal component score—mainly affected by the in-phase pattern—was significantly associated with a large increase in performance at both sets. In contrast, the in-between regions from 80° to 120° were significantly not functional in terms of performance. These findings are consistent with the literature using a dynamical approach to study human coordination (Kelso, 1984; Haken et al., 1985; Kelso, 2021; Zanone and Kelso, 1992; Zanone and Kelso, 1997), which reports that in-phase and out-of-phase constitute stable coordinated states toward which nonlinear coupled oscillators – knee joints here – tend to be naturally attracted. These coordination modes—or *attractors*—emerge from various interacting environmental, task and intrinsic constraints and have also been identified in the postural state space in a supra-postural task involving a visual target to be tracked (Bardy et al., 1999; Bardy et al., 2002). The existence of such in-phase and out-of-phase attractors can justify the self-organized nature of the postural system in which the great dimensionality (i.e., multitude of segments, joints and muscles) must be reduced to make it controllable. In the view of synergetics (Haken, 1987), the information exchanged between the nonlinearly interacting parts of a system can be compressed at the macroscopic level into a collective low-dimensional variable called *order parameter.* In our game, the relative phase variable was sufficient to capture the collective behavior of the left and right knee joints, which was organized into the two macroscopic in-phase and out-of-phase stable states. At the clinical level, this *dimensional reduction* has some relevance because the uninjured and injured knees might be constrained to act as a single unit through our game, and therefore help patients restore their altered perception-action couplings.

Another main finding at this level of analysis lies in the evolution of these preferred coordination patterns with practice. While neither in-phase nor out-of-phase behavior were found to be significantly more or less adopted from the first set to the second set of the experiment, the overall coordination described by both patterns in the PCA space revealed significant differences between the two sets. In other words, the in-phase and out-of-phase patterns were spontaneously selected since the beginning of the game, but the ability to combine them while playing evolves with significant changes. Moreover, it seems that trials with lower performance can be clustered into a coordination mode that could be described neither by a strong in-phase nor a strong out-of-phase component, while a cluster combining both in-phase and out-of-phase seems to be adequate to characterize trials with a better performance. A tendency to move away from the *Low-Performance* cluster to get closer to the *High-Performance* cluster at the end of the experiment was also observed in the PCA space. Taken together, these results can be interpreted on the basis of previous studies analyzing dynamic transitions between coordination modes in learning protocols (Zanone and Kelso, 1992; Zanone and Kelso, 1997) and supra-postural tasks (Bardy et al., 1999; Bardy et al., 2002). In these studies inspired by the Haken-Kelso-Bunz (HKB) model (1985) (Haken et al., 1985; Kelso, 2021), the dynamics of the order parameter can be illustrated in a dynamical landscape defined by a potential function, where behavior is pulled toward attractors of a certain stability represented by wells in the landscape. Postural reorganization can be described either by a qualitative alteration of the landscape—or *bifurcation*—with the emergence of new attractors in the phase space—which thereby becomes multistable—or by a relocation of the attractive states in the landscape—or *shift*. Learning a new motor skill is related to constraints and can be assessed through the study of changes in the dynamical landscape. In the present task, a *bistable* landscape was detected all along the experimental session, with the identification of the two attractors defined by the in-phase and out-of-phase coordination modes.

Significant changes in the PCA score over practice let us suggest that our joint-action game fosters the exploration of the dynamical landscape—i.e., the exploration of a constellation of environmental, task and intrinsic constraints to select coordinative solutions when performing. More specifically, it can be hypothesized that the dynamical landscape of dyads with lower performance was less stable compared to that of dyads with higher performance. Indeed, neither in-phase nor out-of-phase seemed to be particularly apparent in the cluster of the former, while the cluster of the latter was more characterized by these two patterns. Although further analyses are required here, this hypothesis is line with Bernstein’s theory of motor learning (Bernstein, 1967), where the exploration of a wide array of movement solutions is necessary when learning any new motor skill. In contrast, one can expect to observe in the landscape of the most skilled participants abrupt transitions between in-phase and out-of-phase modes, with no intermediate relative-phase values (Bardy et al., 2002).

Finally, relative-phase analyses with the PCA method could be useful in the clinical assessment of patients with postural deficits; postural reorganization in terms of knee–knee coordination changes could be tracked in a potential dynamical landscape. While PCA analysis on knee–knee coordination is a novelty introduced by our study, alterations in inter-joint and inter-segment coordination have already been identified in ACL patients by a few studies employing relative-phase measures (Kurz et al., 2005; Kiefer et al., 2013; Nematollahi et al., 2016; Blache et al., 2016; Park and Yoon, 2021) and/or PCA method (St-Onge et al., 2004; Smeets et al., 2020). Such alterations manifest themselves as rearranged coordination patterns on injured and uninjured legs after ACL rupture (St-Onge et al., 2004), impaired foot-shank and shank-thigh relative-phase dynamics during walking and running (Kurz et al., 2005) as well as stepping (Nematollahi et al., 2016), disrupted coupling between ankle and hip joints in a supra-postural visual-tracking task (Kiefer et al., 2013; i.e., the task that Bardy et al. (1999) designed earlier to demonstrate that postural modes were emergent phenomena), excessive asymmetry in inter-joint coordination in single-leg jump (Blache et al., 2016), protective task-independent single-joint alterations and task-dependent (i.e., landing) whole-body alterations during return-to-sport (RTS) (Smeets et al., 2020). In addition, Park and Yoon (2021) warned on the undesirable consequences of the altered coordination found in ACL patients during a cutting manoeuvre. For instance, the excessive rigidity found in hip-knee coordination—i.e., lower relative-phase variability compared to healthy controls—can be interpreted as a pathological behavior, because it reduces the postural system adaptability to perturbations and because the repetition of a single coordination pattern exerts persistent load on a restricted area, which can lead to structural damages and functional complications on the long-term period. As another example, the abnormally great in-phase pattern that the authors found in the knee-ankle coordination of ACL patients increases the risk of re-injury, due to an exaggerated knee valgus combined with ankle inversion when the coordinated movements of these joints are executed in the same direction. These illustrative scenarios of excessively rigid coordination patterns highlight the need of developing coordinative solutions during ACL rehabilitation. In light of these considerations, it is also worth noting that patients with knee injuries may adopt maladaptive patterns or rely on upper-limb movements rather than exploring knee coordinative solutions. These adaptations can potentially lead to undesirable consequences in rehabilitation. Therefore, we suggest that expanding the scope of future research to include additional joints beyond the knee could provide further insight into the potential clinical benefits of our approach.

To our knowledge, the identification of coordination patterns between the left and right knees during a supra-postural task has not yet been addressed in ACL research. In gait analysis, only one study—motivated by applying the HKB model in practice—investigated the coupling strength between the injured and uninjured knees of ACL patients (Armitano et al., 2018). It was found that ACL patients exhibited lower coordination stability compared to healthy controls, which the authors interpreted as decreased coupling strength between lower limbs while walking. Using the HKB framework, we argue that such a loss of stability may be functional to explore and re-learn coordination patterns. The present study suggests a new potential approach for rehabilitation settings, using PCA to monitor the evolution of knee–knee coordination patterns during an intervention. Moreover, it can be hypothesized that the landscape exploration in our joint-action game could help minimize the production of undesirable rigid repeated patterns in patients with knee injury, while supporting the discovery of new coordinative solutions.

### 4.2 Interpersonal synergies

Although the hypothesis that interpersonal synergies would emerge by the end of practice was not verified, the UCM ratio significantly increased from the first set to the second set of the session. This indicates that the coordination of the two players’ movements evolved in such a way that the board center height became significantly more stabilized with practice.

It is worth noting that individual characteristics such as height and weight were not controlled within the pairs, although these factors may have influenced the collective behavior in the joint-action task. Asymmetries in anthropometric (and other) individual factors can result in asymmetries in the spatiotemporal patterning of co-actors’ movements. In a complementary joint-action task, Richardson et al. (2015) demonstrated that emergent asymmetries in individual behaviors could enhance collective success. While examining the influence of asymmetric individual characteristics on movement patterns would certainly be interesting, our study primarily focused on investigating whether individual movement patterns could be structured through practice to facilitate the emergence of interpersonal synergy in the joint-action game. Future research is encouraged to explore how anthropometric asymmetries affect synergy formation and, in clinical contexts, how inherent asymmetries within injured-uninjured dyads may influence this process.

The fact that UCM scores remained below 1 on average in the later session might be attributed to insufficient practice. Since performance improvement was not observed in all dyads, it could be suggested that those dyads who did not achieve better scores by the end of the session were likely still exploring different game strategies. This is particularly plausible given the nature of the task, which was both new and complex, involving multiple degrees of freedom at inter-limb and inter-player coordination levels and requiring a combination of precision and balance abilities.

Furthermore, the significant correlation between an increase in performance and an increase in the UCM ratio suggests that interpersonal synergies may have become more prominent with a longer period of practice. In the present study, the unstructured variability in the participants’ hand movements can be interpreted as exploratory behavior aimed at finding effective interpersonal coordinative solutions.

Moreover, this inter-agent coordination significantly differed in trials with lower performance and in trials with higher performance. Higher performance was characterized by the emergence of interpersonal synergies. The emergence of interpersonal synergies through our game may suggest that our joint-action hypothesis could actually turn to be a new promising approach on rehabilitation settings. By virtue of its inherent principles of *dimensional reduction* and *reciprocal compensation*, interpersonal synergy through joint action should encourage patients to re-learn perception-action by “moving just as one” with their healthy teammate, whose the structural and behavioral complexity is intact. The idea of restoring complexity in pathological individuals through interpersonal coordination trainings with healthy pairs has already been explored by testing the Complexity Matching hypothesis (West et al., 2008) as an original rehabilitation technique. This hypothesis – according to which a pathological (decomplexified) system could restore its complexity by maximizing information exchange with a healthy (complex) system – was particularly applied into the design of trainings where younger and older agents are mechanically coupled (i.e., arm-in-arm walking), which deliver convincing results in terms of postural stability improvement (Almurad et al., 2018; Ezzina et al., 2021). Although it is interesting to test the efficiency of this Complexity Matching training in pathological populations, side-by-side coupling does not beneficiate from the shared-goal nature of joint action. For this reason, it can be hypothesized that joint action would more effectively foster the emergence of interpersonal synergies and therefore maximize the valuable information exchange between pathological and healthy co-agents through reciprocal compensation.

While the implementation of joint action in clinics may bring additional value to rehabilitation as explained above, some authors also warned on the necessity of providing a smart dyadic task design for avoiding the patient’s disengagement—i.e., slacking behavior—even when the overall performance is maintained (Mace et al., 2017). In their joint-action task involving a stroke patient and a healthy partner, a virtual beam—whose each extremity height is controlled by a player—has to be inclined so that the virtual ball on it reaches a displayed sequence of target of various heights. At the same time, the participants must keep the ball from rolling off the beam. The combination of these two goals to successfully achieve the task inevitably disallows any slacking behavior, since the beam inclination induced by one player to reach a target needs to be adjusted by the second player to keep the ball balanced on the horizontal beam. Such constraints might inspire an updated version of our game. For now, our findings in uninjured dyads suggest that both players co-adjust the board extremities’ height so that the board center height is stabilized, while the ball keeps reaching targets.

Finally, the practical significance of the present study can be highlighted by noting that the applicability of our game in a clinical setting was a key consideration during its manufacturing process. The game was specifically designed to explore joint action as a novel approach to rehabilitation. Consequently, the technical solutions and materials used in its assembly were chosen to meet key functional requirements, including usability (i.e., appropriate dimensions, lightness, resistance to compression and traction), simplicity (i.e., requiring only one additional piece of equipment alongside the BOSU, with straightforward game rules) and affordability (i.e., exclusively inexpensive materials). This careful design approach ensures that our joint-action game is accessible for therapeutic applications and may encourage the development of further research on its clinical relevance.

## 5 Conclusion

In conclusion, the present study provides evidence that our joint-action game fosters the exploration of coordinative solutions in healthy individuals, leading to a convergence toward stable coordination patterns. Moreover, synergy formation was observed at the interpersonal level when higher performance was reached. Together, these findings suggest that our game designed from a systems perspective may be beneficial from a clinical standpoint, through the aspects of exploration, dimensional reduction and reciprocal compensation that it supports. To our knowledge, the joint-action hypothesis had never been tested before as a potential rehabilitation technique using coordination and synergy analyses. Further research is encouraged to test our hypotheses in a population with knee injury.

## Data Availability

The raw data supporting the conclusions of this article will be made available by the authors, without undue reservation.
