# Supplementary material for: Inter-limb and inter-agent coordination in an original joint-action game: exploring novel approaches for clinical practice
Source: Front Psychol. 2025 Mar 24;16:1514957. doi: 10.3389/fpsyg.2025.1514957 (PMC11973364; doi:10.3389/fpsyg.2025.1514957)
Supplement: Supplementary file 1 [file Supplementary_file_1.pdf]

Appendix A

Detailed methodology based on linear multiple regression used to compute the UCM scores.

In each trial, the UCM was analysed over the dataset of time points t varying from frame 1 to frame N = 1680. At each time point t, the EVs (or task-relevant elements) defined as the hands height of the first and second participants of the dyad studied – respectively, h_i and h_j, were given by the vector **T** of dimension n = 2, as following:

$$\mathbf{T}^t = \begin{bmatrix} h_i^t \\ h_j^t \end{bmatrix}$$

For the PV defined as the board center height h_c, the corresponding vector **p** of dimension d = 1 was given at each time point t by:

$$\mathbf{p}^t = [\, h_c^t \,]$$

The reference configuration corresponds to the mean values over the trial of the EVs and PV – respectively $\mathbf{T}^0$ and $\mathbf{p}^0$, given by:

$$\mathbf{T}^0 = \begin{bmatrix} h_i^0 \\ h_j^0 \end{bmatrix}$$

$$\mathbf{p}^0 = [\, h_c^0 \,]$$

At each time point t of the trial, linear approximations were assumed between small changes in magnitude of the EVs and PV with respect to the reference configuration. Linear approximations were based on a Jacobian matrix $\mathbf{J}(\mathbf{T}^0)$ and given at each time point t by Equation 1:

$$\mathbf{p}^{\mathrm{t}} - \mathbf{p}^0 = \mathbf{J}(\mathbf{T}^0)\,.\,(\mathbf{T}^t - \mathbf{T}^0) \quad \textbf{(Equation 1)}$$

The Jacobian matrix $\mathbf{J}(\mathbf{T}^0)$ was estimated using the linear multiple regression method based on the methodology presented by Klous and colleagues (Klous et al., 2010), as shown in Equation 2:

$$(h_c^t - h_c^0) = K_1.\left(h_i^t - h_i^0\right) + K_2.\left(h_j^t - h_j^0\right) \quad \textbf{(Equation 2)}$$

This linear multiple regression was computed from the complete 1680-length dataset. The estimated coefficients of regression K_1 and K_2 correspond to the entries of the Jacobian matrix $\mathbf{J}(\mathbf{T}^0)$ at the reference configuration for each trial, as following:

$$\mathbf{J}(\mathbf{T}^0) = [K_1 \quad K_2]$$

To assess multicollinearity issues when computing the linear multiple regression, the variance inflation factor *VIF* was used to estimate how magnified was the variance of the coefficients of regression because of linear dependence between the EVs (Allison, 1999). The *VIF* was calculated using the coefficient of determination r^2 from the linear multiple regression, according to Equation 3. Higher values of *VIF* reveal higher correlations among EVs which lead to unreliable estimates of the coefficients of regression K_1 and K_2 and therefore to unreliable UCM results.

$$VIF = 1/(1 - r^2) \quad \textbf{(Equation 3)}$$

The UCM subspace was approximated with the null-space of the Jacobian matrix $\mathbf{J}(\mathbf{T}^0)$ that contains all the EVs combinations that stabilize the PV. Because the difference between the dimensions of the vectors **T** (i.e., n = 2) and **p** (i.e., d = 1) equals to 1, the null-space was obtained by computing the one-basis vector ε_i where $i = n - d = 2 - 1 = 1$, (with the Matlab function *null*), solving Equation 4:

$$0 = \mathbf{J}(\mathbf{T}^0) \,.\, \varepsilon_i \quad \textbf{(Equation 4)}$$

The vector $(\mathbf{T}^t - \mathbf{T}^0)$ of the deviations of the EVs from the reference configuration was resolved into its projections $f_{\|}$ and $f_{\perp}$ along and orthogonal to the UCM subspace, respectively, according to Equation 5 and Equation 6:

$$f_{\|} = \sum_{i=1}^{n-d} (\,\varepsilon_i^T . (\mathbf{T}^t - \mathbf{T}^0)) \,.\, \varepsilon_i \quad \textbf{(Equation 5)}$$

$$f_{\perp} = (\mathbf{T}^t - \mathbf{T}^0) - f_{\|} \quad \textbf{(Equation 6)}$$

The variance along the UCM subspace V_{UCM} (i.e., which stabilizes the PV) and the variance orthogonal to the UCM subspace V_{ORT} (i.e., which destabilizes the PV) were normalized by the number of DoF of their respective subspace and were respectively calculated by Equation 7 and Equation 8:

$$V_{UCM} = \sigma_{\|}^2 = \frac{1}{(n-d).N} \sum_{i=1}^{N} f_{\|}^2 \quad \textbf{(Equation 7)}$$

$$V_{ORT} = \sigma_{\perp}^2 = \frac{1}{d.N} \sum_{i=1}^{N} f_{\perp}^2 \quad \textbf{(Equation 8)}$$

For each trial, the final UCM score that indicates the presence or absence of synergy is obtained by comparing the magnitudes of V_{UCM} and V_{ORT}, using the ratio *UCM* of Equation 9:

$$UCM = \frac{V_{UCM}}{V_{ORT}} \quad \textbf{(Equation 9)}$$

References

Allison PD. Multiple regression: A primer. Pine Forge Press. Thousand Oaks, Calif. 1999.

Klous M, Danna-dos-Santos A, Latash ML. Multi-muscle synergies in a dual postural task: evidence for the principle of superposition. Exp Brain Res. 2010 Apr;202(2):457–71.
