# Supplementary figures and images for: Inter-limb and inter-agent coordination in an original joint-action game: exploring novel approaches for clinical practice
Source: Front Psychol. 2025 Mar 24;16:1514957. doi: 10.3389/fpsyg.2025.1514957 (PMC11973364; doi:10.3389/fpsyg.2025.1514957)

## Appendix B

Evolution of the performance scores from Set 1 to Set 2 in each dyad (but Dyad 10).

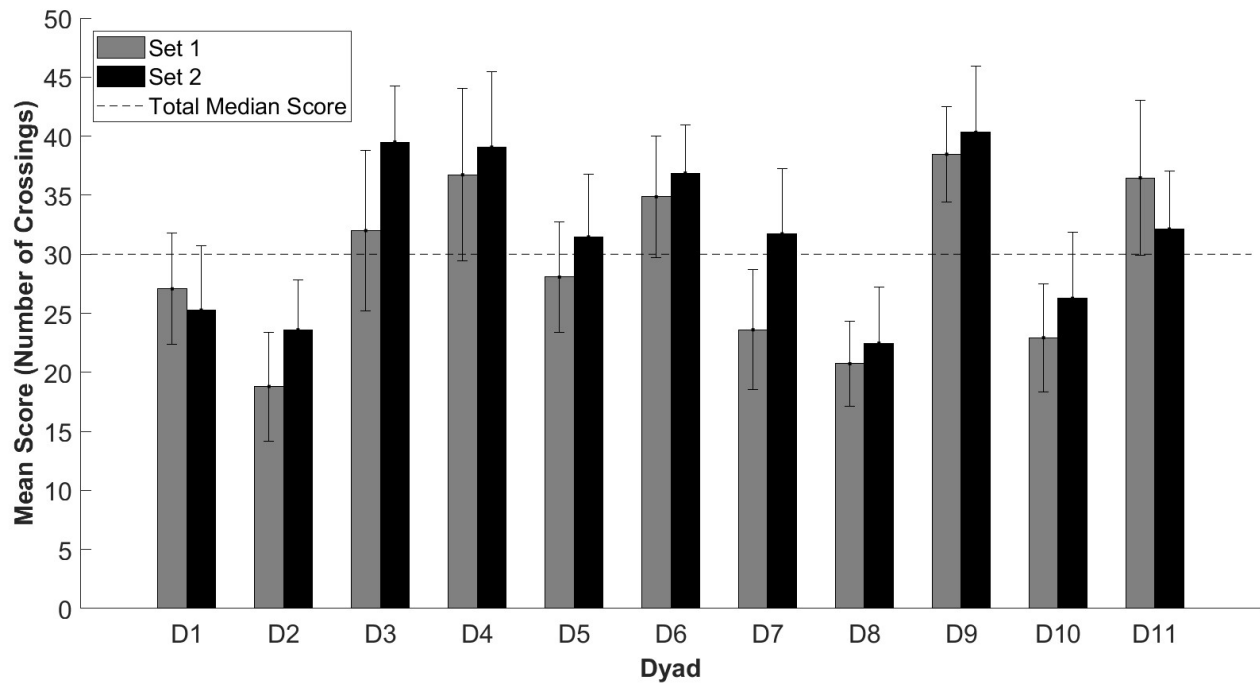

Supplement: Supplementary file 2 [file Supplementary_file_2.pdf]
